# Editorial: Tissue repair modeling of locomotor apparatus

**DOI:** 10.3389/fcell.2025.1653787

**Published:** 2025-09-19

**Authors:** Magali Cucchiarini, Karel Dorey, Ana Rey Rico, Serena Duchi

**Affiliations:** ^1^ Center of Experimental Orthopaedics and Osteoarthritis Research, Saarland University Medical Center, Homburg, Germany; ^2^ Division of Developmental Biology and Medicine, School of Medical Sciences, Faculty of Biology, Medicine and Health, University of Manchester, Manchester, United Kingdom; ^3^ Gene & Cell Therapy Unit (G-CEL), Centro Interdisciplinar de Química e Bioloxía – CICA, Universidade da Coruña, Coruña, Spain; ^4^ Department of Surgery, The University of Melbourne and Aikenhead Center for Medical Discovery (ACMD) @ St. Vincent’s Hospital, Fitzroy, Melbourne, Victoria, VIC, Australia

**Keywords:** locomotor apparatus, muscoloskeletal system, cartilage repair, biomimetic scaffolds, epigenetics, cell therapeutic products, bone regeneration, mesenchymal stem cells

## 1 Introduction

The human locomotor apparatus—a complex system comprising bones, cartilage, muscles, tendons, ligaments, and neural signaling—serves as the foundation of human movement (Sarkodie-Gyan and Yu, 2023). Injuries and degenerative conditions affecting this system are common and often lead to substantial impairments in mobility and diminished quality of life. Given the increasing incidence of musculoskeletal disorders due to aging populations, sports-related injuries, and chronic conditions such as osteoarthritis, research into tissue repair modeling has never been more crucial (Gill et al., 2023). Advancements in this field offer promising avenues to enhance our understanding and treatment for these disorders (Vernengo et al., 2023; Chehade et al., 2020).

**FIGURE 1:**
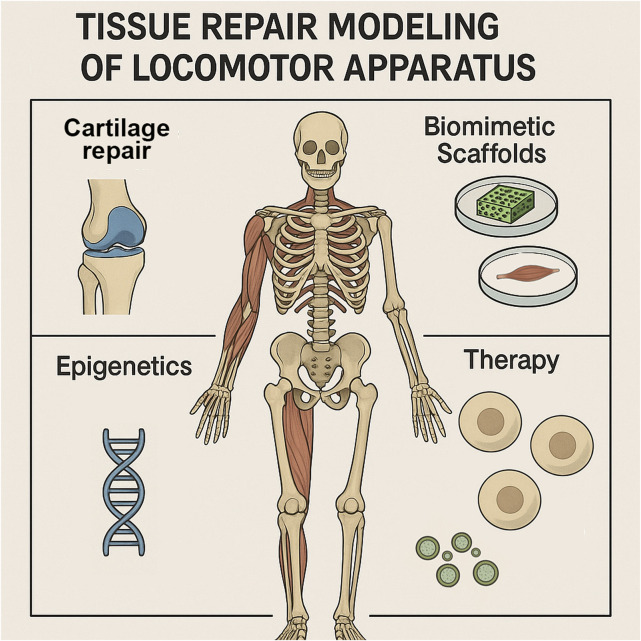
Graphical abstract illustrating the themes of this editorial on tissue repair modeling of locomotor apparatus. Highlighted areas include cartilage repair, biomimetic scaffolds, epigenetic regulation, and therapeutic strategies, which together represent the interdisciplinary approaches discussed in this Research Topic to advance understanding and treatment of human musculoskeletal disorders.

This Research Topic compiles eight insightful articles that delve into various aspects of tissue repair within the locomotor apparatus (Figure 1). These studies explore innovative modeling approaches, advanced biomaterials, therapeutic strategies, and the intricate molecular mechanisms underlying tissue regeneration. The findings presented here underscore the importance of interdisciplinary research in bridging the gap between fundamental science and clinical applications, paving the way for more effective and personalized treatment modalities.

## 2 Innovative model for cartilage repair

Cartilage injuries present a significant challenge due to the tissue’s limited regenerative capacity and lack of direct vascularization. Trengove et al. introduced a dynamically loaded *ex vivo* model to study neocartilage formation and integration in human cartilage repair. This model replicates the mechanical environment of cartilage, allowing researchers to investigate the effects of mechanical stimulation on engineered tissue constructs. The study’s findings underscore the importance of mechanical stimuli in promoting neocartilage formation and integration, reinforcing the necessity of biomechanical factors in cartilage tissue engineering. By providing a controlled environment for testing cartilage repair strategies, this model may contribute to the development of more effective therapies for joint injuries and osteoarthritis.

## 3 Advancements in biomimetic scaffolds

Scaffolds that mimic the native extracellular matrix (ECM) of bone tissue play a critical role in tissue engineering. Doyle et al. assessed the impact of geometrical design on the stiffness and angiogenic potential of 3D-printed bone-mimicking scaffolds. Their research highlights how specific structural parameters influence both the mechanical properties and biological performance of scaffolds. By optimizing scaffold design, researchers can create constructs that better support cell adhesion, proliferation, and differentiation, ultimately enhancing bone regeneration. The study provides valuable insights for developing personalized scaffolds that cater to patient-specific needs, improving outcomes in orthopedic and reconstructive surgery.

## 4 Molecular insights into enthesis integrity

The fibrocartilaginous enthesis, where tendons attach to bones, is a highly specialized structure that is essential for musculoskeletal function. Yambe et al. examined the role of sclerostin in modulating mineralization and stiffness at the enthesis. Their study reveals that sclerostin plays a critical role in maintaining mechanical tissue integrity by regulating the mineralization processes. Given the high susceptibility of the enthesis to injury, particularly in conditions such as tendinopathy and enthesopathy, understanding the molecular pathways governing its repair is crucial. These findings could inform the development of targeted therapies that enhance enthesis healing and reduce the risk of chronic musculoskeletal disorders.

## 5 Enhancing angiogenesis in tissue engineering

Successful tissue repair relies heavily on the formation of new blood vessels to supply nutrients and oxygen to regenerating cells. Giretová et al. investigated the effect of incorporating an agarose/gelatin gel into polyhydroxybutyrate/chitosan scaffolds to increase their pro-angiogenic potential. The addition of the gel component was found to enhance the scaffolds’ ability to promote blood vessel formation, suggesting a promising approach to improve vascularization in engineered tissues. These findings highlight the importance of developpromising avenues to enhance our understanding of anding bioactive scaffolds that actively support angiogenesis, which is essential for the long-term success of tissue-engineered constructs in bone and soft tissue repair.

## 6 Epigenetic regulation in osteogenesis

The differentiation of mesenchymal stem cells (MSCs) into osteoblasts is a tightly regulated process influenced by genetic and epigenetic factors. In this Research Topic, Lai et al. examined the impact of DNA methylation on the osteogenic differentiation of MSCs in a review article, providing insights into how epigenetic modifications regulate bone formation. Understanding these mechanisms is critical for developing strategies that enhance bone regeneration. By manipulating epigenetic regulators, researchers may be able to improve the efficacy of stem cell-based therapies and develop novel pharmacological interventions for the treatment of osteoporosis and other bone disorders.

## 7 Stem cell therapies for bone regeneration

Stem cell-based approaches hold immense promise for treating complex bone defects. Teixeira et al. reported on the use of bone marrow-derived mesenchymal stem cells (MSCs) to treat children with osteonecrosis secondary to sickle cell disease. Their findings suggest that MSC therapy can promote bone regeneration in this patient population, highlighting the potential of stem cell-based approaches in addressing challenging bone repair scenarios. Given the limited treatment options for osteonecrosis, particularly in pediatric patients, these findings provide a foundation for the future clinical application of MSC therapy in regenerative medicine.

## 8 Small extracellular vesicle therapy in osteoarthritis

A systematic review by González-Rodríguez et al. synthesized research from 2019 to 2025 on the therapeutic and biomarker potential of small extracellular vesicles (sEVs) in osteoarthritis. Focusing on sEVs derived from mesenchymal stem cells, synoviocytes, chondrocytes, and induced pluripotent stem cells, the review highlights their anti-inflammatory and regenerative properties, demonstrating their efficacy in modulating the joint microenvironment, prompting chondrogenesis, and reducing pain across preclinical and early clinical settings. While the fundamental understanding of osteoarthritis pathophysiology remains anchored in well-established mechanisms, such as cartilage degradation, inflammation, and subchondral bone alterations, the review underscores significant progress in sEV-based therapeutic research. It emphasizes critical challenges, including the need for standardized isolation protocols, elucidation of mechanistic pathways, and navigation of regulatory hurdles, as essential next steps to realizing the full potential of sEV therapies for osteoarthritis.

## 9 Muscle regeneration *in vitro*

In their study, Kase et al. introduced a simplified *in vitro* model for skeletal muscle regeneration using primary mouse cells, overcoming the limitations of complex culture systems. The model evaluates macrophage-dependent regeneration in a two-dimensional culture and reveals that endotoxin pre-stimulation alters macrophage gene expression, significantly reducing their contribution to muscle repair. These findings highlight the role of macrophage responsiveness in regeneration efficiency and offer a valuable tool for investigating the contributions of various cell types, such as macrophages and muscle stem cells, to skeletal muscle regeneration.

## 10 Future directions

The articles in this Research Topic deepen our understanding of the intricate processes involved in tissue repair in the locomotor apparatus, emphasizing the need for interdisciplinary approaches that combine biomechanics, molecular biology, materials science, and clinical research to develop more effective therapies. Future research should focus on integrating mechanical and biological factors in tissue engineering, investigating the role of epigenetic regulation in tissue repair, and developing advanced biomaterials that closely mimic the native tissue environment. Translating these findings into clinical applications is also critical and requires collaboration between scientists, engineers, and clinicians to create innovative treatments that restore function and improve the quality of life for individuals with musculoskeletal disorders. Emerging technologies, such as bioprinting, gene editing, and machine learning-driven predictive models, hold great potential to revolutionize the field. As researchers continue to refine therapeutic strategies, the ultimate goal remains to develop personalized solutions that enhance musculoskeletal tissue repair and regeneration, bringing us closer to improving patient outcomes and quality of life for those affected by musculoskeletal conditions.
